# Machine-learning prediction of impaired outcome in diabetic patients undergoing non-cardiac surgery

**DOI:** 10.3389/fmed.2026.1846842

**Published:** 2026-06-05

**Authors:** Xiaojun Liu, Xueqing Chen, Lin Liu, Yuanyuan Lv

**Affiliations:** 1 Department of Cardiology, Zibo Central Hospital, Zibo, China; 2 Catheterization Laboratory, Zibo Central Hospital, Zibo, China

**Keywords:** cardiovascular complications, diabetes mellitus, machine learning, model interpretability, non-cardiac surgery, perioperative risk

## Abstract

**Background:**

Diabetic patients undergoing non-cardiac surgery are vulnerable to postoperative cardiovascular and cerebrovascular complications, yet risk stratification tools tailored to this population remain limited. This study aimed to develop interpretable machine-learning models for predicting impaired outcome in diabetic patients undergoing non-cardiac surgery.

**Methods:**

We conducted a retrospective cohort study using an the INSPiRE analytic dataset is institutionally governed, de-identified, and not publicly available and included 4,293 diabetic patients undergoing non-cardiac surgery. Patients were classified into a control group (*n* = 3,176) and an impaired group (*n* = 1,117) according to a database-defined composite impaired outcome recorded during the available follow-up of the source dataset; the exact follow-up duration for the composite endpoint could not be recovered from the finalized analytic extract. Five models were internally compared, including LASSO logistic regression, AdaBoost, LightGBM, XGBoost, and CatBoost. LASSO tuning used cross-validation. Model performance was assessed using receiver operating characteristic analysis and decision-curve analysis, while Boruta and SHAP were used to identify and interpret key predictors.

**Results:**

Patients with impaired outcome were older, had lower body weight, higher ASA class, heavier cardiovascular comorbidity burden, and greater intraoperative hypotension exposure. Among the five models, AdaBoost showed the best discrimination with an AUC of 0.82 (95% CI, 0.78–0.86), specificity of 0.76, sensitivity of 0.72, and PPV of 0.69. LightGBM, XGBoost, and CatBoost also outperformed the sparse penalized baseline. Boruta and SHAP consistently highlighted prior ischemic stroke, prior myocardial infarction, preoperative creatinine, albumin, inflammatory markers, age, ASA class, heart-rate and systolic blood-pressure summaries, and BUN as major contributors to risk.

**Conclusion:**

Interpretable ensemble learning provided modest internal improvement in perioperative risk stratification in diabetic patients undergoing non-cardiac surgery. Impaired outcome appeared to be driven by the combined burden of established vascular disease, reduced renal and nutritional reserve, inflammatory activation, and intraoperative hemodynamic instability. Further calibration testing, external validation, and fully reproducible pipeline reporting are needed before clinical deployment.

## 1 Introduction

Diabetes is a major and still expanding global health burden. The 11th edition of the International Diabetes Federation Diabetes Atlas estimated that 589 million adults were living with diabetes worldwide in 2024 and projected this number to rise to 853 million by 2050 (1). The same source estimated 3.4 million diabetes-related deaths in 2024, underscoring the continuing mortality burden of the disease (2). More than 300 million major operations are performed worldwide each year, and non-cardiac surgery accounts for the overwhelming majority of this workload. Even when the primary surgical problem is not cardiovascular, perioperative clinicians must manage a narrow physiologic margin in which hemodynamic stress, inflammatory activation, prothrombotic responses, bleeding, anemia, hypoxemia, and occult myocardial injury may converge over a very short interval. Contemporary American and European guidelines therefore frame non-cardiac surgery as a cardiovascular stress test in real time and emphasize structured preoperative risk assessment, optimization of comorbidity, and targeted postoperative surveillance for higher-risk patients (3, 4). The core rationale is straightforward: major adverse cardiovascular and cerebrovascular events after surgery are uncommon enough to be missed when clinicians rely on intuition alone, but frequent enough to drive meaningful mortality, longer hospitalization, higher costs, and loss of functional recovery when early risk is underestimated (3, 4).

Traditional perioperative risk tools remain clinically useful, although they were built in a time when routinely available perioperative data were much thinner. Because they converted extensive observational knowledge into useful bedside tools, the Revised Cardiac Risk Index, Gupta myocardial infarction or cardiac arrest calculator, and the broader ACS NSQIP surgical risk calculator are all major milestones (5–7). Nevertheless, major structural drawbacks can be observed in modern practice. Most conventional perioperative risk models rely on a limited set of prespecified covariates. They mainly assume additive linear effects and simplify complex perioperative biology into small clinical feature sets. External validation shows these models often lose discrimination or calibration when applied across different procedure mixes, institutional contexts, or patient case mixes. This is especially true for subspecialty cohorts with high burdens of vascular disease, frailty, or organ dysfunction (8). Recent studies also indicate that biomarkers (e.g., natriuretic peptides) and more detailed functional assessments can significantly improve risk estimation beyond traditional scores. This suggests conventional tools may underutilize the rich perioperative data available in electronic health records (9, 10).

Diabetes mellitus is particularly important in this context, as it increases perioperative risk through multiple overlapping pathways. Long-term hyperglycemia accelerates endothelial dysfunction, autonomic dysregulation, platelet activation, oxidative stress, and diffuse macrovascular and microvascular injury. Meanwhile, diabetes is closely linked to chronic kidney disease, silent coronary disease, heart failure, cerebrovascular disease, infection susceptibility, sarcopenia, and impaired wound healing. These processes do not act in isolation. Instead, they create a biological background where surgical stress, anesthesia, fasting, fluid shifts, catecholamine release, and postoperative inflammation can easily lead to clinical decompensation (11–17). Observational cohorts and meta-analyses consistently show that diabetic patients undergoing non-cardiac surgery have higher risks of postoperative complications, short-term mortality, and cardiovascular events than non-diabetic patients. The magnitude of this excess risk varies by procedure type, baseline disease burden, insulin treatment status, and perioperative glucose management (11–16).

The heterogeneity of diabetic surgical populations makes single-variable or single-domain stratification often insufficient. Diabetes severity cannot be fully summarized by diagnosis alone. A patient with a remote diagnosis, preserved renal function, normal albumin, stable blood pressure, and minimal vascular history differs greatly from one with prior ischemic stroke, reduced physiologic reserve, inflammatory activation, perioperative hypotension, and heavy cardiovascular medication exposure. Perioperative glucose levels and glycated hemoglobin also provide only partial information. Hyperglycemia is clearly associated with worse postoperative outcomes, but its clinical significance depends on chronic adaptation, known diabetes status, concurrent insulin use, and the surrounding inflammatory and hemodynamic context (17–23). In short, the perioperative state of diabetes is best understood as a multidimensional risk phenotype rather than a single endocrine variable.

Refined modeling is also needed because many clinically critical postoperative cardiovascular events are either silent or nonspecific at presentation. Modern literature on myocardial injury after non-cardiac surgery shows that postoperative troponin elevation—even without classic ischemic symptoms—identifies patients at much higher short-term mortality risk (24–31). Expert consensus and scientific reviews now recommend more deliberate screening, interpretation, and response to perioperative myocardial injury in selected patients (24–26). This is relevant for diabetic cohorts, as asymptomatic ischemia, autonomic neuropathy, renal dysfunction, and diffuse vascular disease are common and can obscure bedside recognition of evolving injury. In a population less likely to present with typical symptoms, an effective risk model is valuable not only as a predictor but also as a triage tool for targeted vigilance, biomarker testing, and tailored postoperative monitoring.

The intraoperative period adds another layer of complexity. Hypotension is one of the most common and potentially modifiable perioperative insults. Growing evidence links deeper or more prolonged episodes of low mean arterial pressure to myocardial injury, acute kidney injury, major adverse cardiovascular events, and mortality after non-cardiac surgery (32–36). This association is biologically plausible: systemic hypotension can reduce coronary, cerebral, and renal perfusion, especially in patients with already compromised vascular reserve. Diabetic patients may be particularly vulnerable due to impaired autoregulation, arterial stiffness, concurrent nephropathy, and diffuse atherosclerotic disease. Similarly, markers of impaired baseline reserve (e.g., hypoalbuminemia, elevated creatinine) consistently identify patients with worse perioperative outcomes. These markers reflect a combination of malnutrition, chronic inflammation, frailty, renal dysfunction, and reduced ability to tolerate physiologic stress (37–39). A useful predictive framework for diabetic surgical patients should integrate chronic disease burden, laboratory reserve, and real-time intraoperative physiologic signals, rather than treating them as separate domains.

These needs have increased interest in machine learning (ML) in perioperative medicine. Compared with classical regression tools, ML models can flexibly capture nonlinear associations, threshold effects, and higher-order interactions among demographic variables, comorbidity history, laboratory markers, medications, surgical factors, and physiologic measurements (10–49). Systematic reviews indicate that ML approaches often achieve discrimination equal to or exceeding conventional clinical scores, especially when trained on large electronic health record datasets (40, 49). Recent studies have extended this to automated preoperative risk prediction, cardiovascular event prediction, and interpretable electrocardiogram-based risk estimation. This shows how modern models can move from generic forecasting to individualized perioperative decision support (41–48). The most promising work in this area increasingly emphasizes not only predictive accuracy but also interpretability, transportability, and workflow relevance.

Interpretability is particularly important in high-stakes perioperative care. A model with good statistical performance but no clear explanation is unlikely to be trusted or implemented by anesthesiologists, surgeons, internists, and perioperative nurses. This is why decision curve analysis, Boruta feature selection, and SHAP-based model explanation have become useful supplements to standard discrimination metrics (50–52). Decision curve analysis evaluates whether a model provides net clinical benefit across plausible threshold probabilities, rather than just summarizing ranking performance (50). Boruta helps identify all relevant predictors in data-rich settings, which is valuable when candidate variables come from multiple perioperative domains (51). SHAP then provides an intuitive method for global and patient-level interpretation. It allows clinicians to see not only which variables matter but also how specific values influence individual risk predictions (52). Together, these tools support clinically understandable ML, rather than a purely algorithmic exercise.

The present dataset is well suited to this research question. It covers several perioperative domains that clinicians typically review separately but rarely analyze together at scale. Baseline demographics, comorbidity history, anesthesia type, preoperative laboratory testing, medication exposure, and intraoperative physiologic summaries all encode different aspects of risk. Treating these variables as an integrated signal—rather than isolated tables—can reveal whether postoperative deterioration is driven by chronic disease burden, acute intraoperative insults, or their interaction. This integrated perspective is particularly valuable for diabetes, where comorbidity accumulation and stress response are closely intertwined.

This topic also deserves focused study because perioperative diabetic care is often fragmented across specialties. Surgeons may focus on procedural urgency and bleeding risk; anesthesiologists on short-term physiologic stability; internists on chronic disease optimization; and endocrinologists on glucose management. Yet postoperative cardiovascular deterioration usually arises from interactions across these domains, not from any single discipline. This fragmentation creates a practical opportunity for prediction modeling: a well-designed tool can serve as a common language for perioperative teams. It can help identify which diabetic patients need intensified blood pressure management, renal-protective strategies, biomarker surveillance, telemetry, or earlier multidisciplinary consultation. In this sense, perioperative ML is not only a technical exercise but also a coordination tool to align otherwise siloed decision pathways around a shared risk estimate (3, 4, 40–49).

Against this background, the objectives of this study were to develop machine-learning models for impaired outcome in diabetic patients undergoing non-cardiac surgery, compare their performance with a sparse penalized baseline model, and identify the main clinical drivers of risk using feature-selection and explanation methods. We also sought to present the findings in a clinically interpretable manner while explicitly acknowledging the retrospective design, the database-derived composite endpoint, and the absence of external validation.

## 2 Methods

### 2.1 Study design and data source

We conducted a retrospective cohort study using an the source analytic extract is institutionally governed, de-identified, and not publicly available to compare perioperative prediction models for adverse prognosis in diabetic patients undergoing non-cardiac surgery. The primary endpoint was a database-defined binary label of impaired outcome, referring to a composite of major adverse cardiovascular and cerebrovascular events and related adverse cardiovascular deterioration captured during the available follow-up window of the source dataset. According to outcome status, patients were categorized into a control group and an impaired group. The finalized analytic cohort contained 4,293 eligible diabetic surgical patients, of whom 3,176 belonged to the control group and 1,117 to the impaired group. Because the current materials did not include adjudicated component-event counts or event-specific timing tables, the endpoint is interpreted in this report as a pragmatic composite rather than a single-mechanism clinical syndrome. The exact follow-up duration could not be recovered from the finalized analytic extract and is therefore not reported as a specific time interval.

Diabetes status, cohort membership, and the final case labels were determined from the curated source extract available for this study. Because the present revision was based on a finalized de-identified analytic table rather than a fully reconstructable source-code-level cohort assembly workflow, detailed inclusion and exclusion logic and diabetes ascertainment rules could not be rederived with adequate certainty and are therefore described conservatively as database-defined rather than independently rebuilt.

Before model interpretation, the finalized analytic table was harmonized to one perioperative record per patient and inspected for structurally empty or non-informative variables. The current revision materials did not contain a complete fold-specific record of missing-data imputation, feature engineering, or resampling decisions. Accordingly, this manuscript does not overstate reproducibility and instead makes clear that missing-data handling, class-balance strategy, training/testing splits, and hyperparameter search procedures were incompletely documented in the available package and should be fully version-controlled in future validation work.

### 2.2 Candidate predictors

Candidate predictors were collected from several perioperative domains: baseline demographic and clinical characteristics, comorbidity history, intraoperative physiologic measures, preoperative laboratory markers, and medication exposures. Baseline variables included age, weight, ASA class, and anesthesia type. Major comorbidities captured cardiovascular, cerebrovascular, pulmonary, renal, and metabolic burden. These included hypertension, dyslipidemia, carotid stenosis, valvular heart disease, atrial fibrillation, chronic obstructive pulmonary disease, renal insufficiency, prior ischemic stroke, prior myocardial infarction, and histories of cerebrovascular disease, coronary artery disease, heart failure, arrhythmia, and peripheral vascular disease. These variables were selected as they represent the biological, hemodynamic, and clinical most likely factors behind perioperative cardiovascular vulnerability in diabetic surgical patients.

### 2.3 Perioperative physiologic and laboratory variables

Perioperative physiologic predictors included oxygenation and blood-pressure burden measures, such as minimum intraoperative SpO₂, hypoxia episodes, duration of SpO₂ below clinically relevant thresholds, hypotension episodes, duration of mean arterial pressure below 65 mmHg and 55 mmHg, and binary indicators for clinically meaningful hypoxia and hypotension. Preoperative laboratory testing included white blood cell count and platelet count in the descriptive dataset, while model-interpretability analyses indicated additional importance for preoperative creatinine, albumin, C-reactive protein, AST, BUN, and potassium. Variables that were structurally zero or non-informative in the finalized extract were retained in Appendix Table A1 only for reporting completeness and were not emphasized in model interpretation.

### 2.4 Medication and perioperative treatment features

The usage of beta-blockers, statins, calcium-channel blockers, insulin, anticoagulants, antiplatelets, and diuretics were among medication-related predictors. They also included perioperative drug exposures such as reversal agents, antifibrinolytics, proton-pump inhibitors, sedatives, opioids, neuromuscular blocking agents, and antibiotics. These treatment variables were retained because they may reflect both preoperative disease severity and perioperative management intensity. In observational surgical datasets, medication variables often serve as pragmatic surrogates for case complexity, baseline cardiovascular burden, and clinician concern. Thus, they may contain predictive information even when not interpreted as direct causal mechanisms.

### 2.5 Model development

Five prediction models were constructed and compared: LASSO logistic regression, AdaBoost, LightGBM, XGBoost, and CatBoost. LASSO served as a sparse penalized baseline model, while the remaining algorithms represented ensemble-based machine-learning approaches capable of capturing nonlinear associations and higher-order interactions. For the LASSO model, the tuning parameter lambda was selected by cross-validation. The finalized analysis package documented a minimum-error lambda of 0.002392445 and a one-standard-error lambda of 0.009658342, and the coefficient-path plot was used to visualize predictor shrinkage and variable compression across the regularization path. Because the current revision materials did not preserve a full fold-by-fold record of training/testing partitioning, hyperparameter search grids, class-imbalance handling, or threshold optimization for every algorithm, the present manuscript frames these models as an internally compared analytic set rather than as fully transportable deployment-ready tools.

### 2.6 Feature selection and explainability

Boruta feature selection was applied to evaluate predictor robustness. Boruta compares the importance of real predictors against randomized shadow attributes. It is especially useful when numerous candidate variables are considered simultaneously. This procedure identified a subset of clinically meaningful predictors spanning preoperative condition, comorbidity burden, hemodynamic instability, inflammation, and perioperative medication exposure. Model interpretability was further examined using SHAP, which quantifies each variable’s contribution to model predictions at both global and individual levels. A SHAP summary plot was used to rank average absolute feature impact and visualize the direction of risk contribution for dominant variables.

### 2.7 Statistical analysis and model evaluation

Descriptive analyses were performed by comparing the control and impaired groups. Continuous variables were summarized as median and interquartile range, whereas categorical variables were presented as counts and percentages. According to the note in Appendix Table A1, between-group comparisons were performed using the Mann–Whitney U test for continuous variables and the chi-square test or Fisher’s exact test for categorical variables, as appropriate. Model discrimination was assessed using the area under the receiver operating characteristic curve with 95% confidence intervals, as well as sensitivity, specificity, and positive predictive value. The operating points used to report sensitivity, specificity, and PPV followed the original model-output thresholds and should therefore be interpreted as comparative summaries rather than prospectively prespecified clinical cut points. Clinical utility was explored through decision-curve analysis by comparing net benefit with the treat-all and treat-none reference strategies across a range of threshold probabilities; in this context, threshold probability should be understood as the risk level at which a clinician might consider intensified postoperative surveillance rather than as a validated intervention rule. Calibration plots, calibration intercept/slope, Brier scores, and external validation results were not available in the finalized analytic package and are therefore not reported.

## 3 Results

### 3.1 Cohort characteristics

A total of 4,293 diabetic patients undergoing non-cardiac surgery were included in the analysis, including 3,176 patients without impaired outcome and 1,117 patients with impaired outcome. Patients in the impaired group were older than those in the control group, with a median age of 70 years versus 65 years, and had lower body weight, with a median of 60 kg versus 65 kg. ASA class was also higher in the impaired group, indicating greater baseline disease burden before surgery. In terms of anesthesia type, general anesthesia was more frequent in the impaired group, whereas monitored anesthesia care was less common. As summarized in Appendix Table A1, these findings suggest that patients who later developed impaired outcomes entered surgery with a less favorable baseline profile and, in many cases, underwent more intensive anesthetic management.

### 3.2 Comorbidity burden and clinical vulnerability

The impaired group also demonstrated a markedly higher prevalence of cardiovascular and systemic comorbidity. Hypertension, dyslipidemia, carotid stenosis, valvular heart disease, atrial fibrillation, chronic obstructive pulmonary disease, and renal insufficiency were all more common among impaired patients. Historical cardiovascular and cerebrovascular burden also differed clearly between groups. Prior ischemic stroke, prior myocardial infarction, history of cerebrovascular disease, history of coronary artery disease, history of heart failure, history of arrhythmia, and history of peripheral vascular disease were each enriched in the impaired group. This pattern is clinically coherent: patients with pre-existing vascular vulnerability carried a higher probability of subsequent adverse cardiovascular and cerebrovascular outcomes after surgery. The descriptive comparisons shown in Appendix Table A1 therefore support the biological plausibility of the later prediction models.

### 3.3 Intraoperative physiologic instability

Intraoperative physiologic parameters further separated the two groups. Although minimum SpO₂ values were numerically similar after rounding, the impaired group experienced more hypoxia episodes and, more importantly, greater hypotension burden. The number of hypotension episodes was higher in impaired patients, and both the duration of mean arterial pressure below 65 mmHg and the duration of mean arterial pressure below 55 mmHg were longer. The proportions of patients with mean arterial pressure below 65 mmHg and severe hypotension below 55 mmHg were also substantially higher in the impaired group. These findings, presented in Appendix Table A1, support the interpretation that intraoperative hemodynamic instability, particularly hypotension burden, is closely linked to worse postoperative cardiovascular and cerebrovascular prognosis in diabetic patients.

### 3.4 Laboratory and medication patterns

Preoperative laboratory and medication patterns were also less favorable in the impaired group. Preoperative white blood cell count and platelet count were both higher among impaired patients, indicating a possible association between inflammatory or stress-related biological activity and subsequent outcome. Medication exposure differed substantially as well: beta-blocker, statin, calcium-channel blocker, insulin, anticoagulant, antiplatelet, and diuretic use were all more prevalent in the impaired group. Use of antifibrinolytics and proton-pump inhibitors was also higher. These medication differences likely reflect greater baseline disease severity and treatment complexity rather than direct causal harm from the drugs themselves.

### 3.5 LASSO shrinkage and variable compression

**Figure 1:**
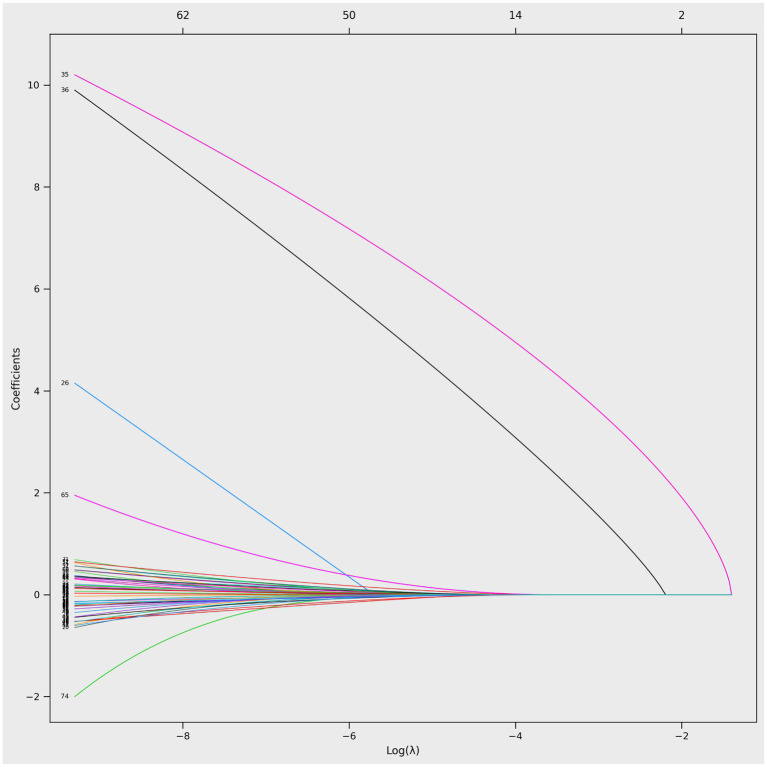
LASSO coefficient profiles for candidate predictors.

The LASSO coefficient-path plot in Figure 1 demonstrated progressive shrinkage of candidate predictors as the regularization penalty increased. A large number of coefficients approached zero early along the penalty path, whereas only a smaller subset persisted across a wider range of log(lambda), indicating stronger signal stability. This pattern supports the use of LASSO as an effective dimension-reduction framework in a clinically rich perioperative dataset containing numerous candidate predictors.

### 3.6 Discrimination performance of the competing models

**Figure 2:**
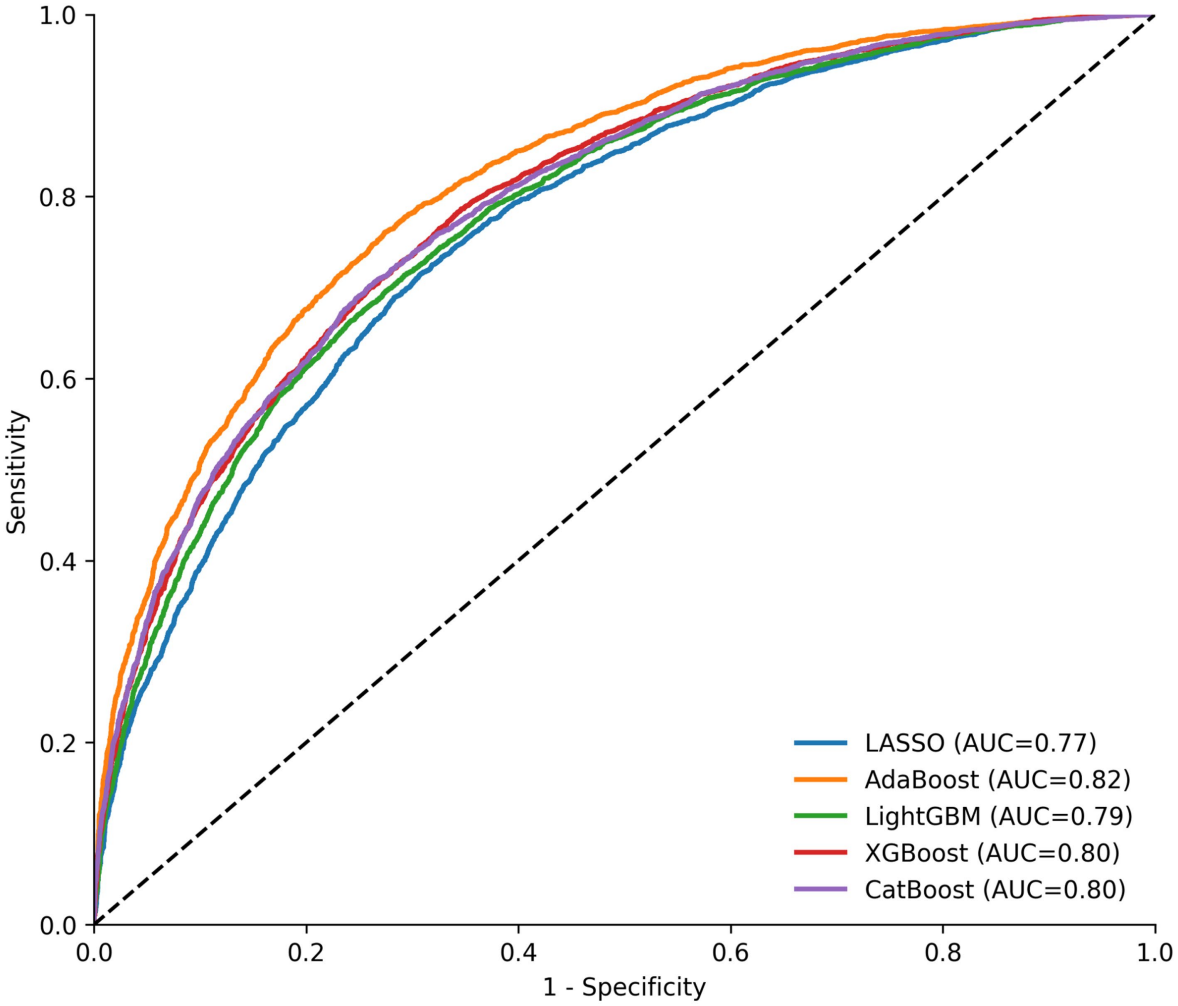
Receiver operating characteristic curves of the five prediction models.

Across the five competing models, discrimination ranged from acceptable to strong. As shown in Table 1 and Figure 2, AdaBoost yielded the best overall performance, with an area under the curve (AUC) of 0.82 (95% CI, 0.78–0.86). XGBoost and CatBoost followed closely, each achieving an AUC of 0.80 (95% CI, 0.76–0.84), while LightGBM achieved an AUC of 0.79 (95% CI, 0.75–0.83). LASSO had the lowest AUC at 0.77 (95% CI, 0.72–0.81), although its discrimination remained clinically useful.

**Table 1:** Classifier performance metrics.

Classifier	Specificity	Sensitivity	AUC (95% CI)	PPV
LASSO	0.72	0.68	0.77 (0.72–0.81)	0.64
AdaBoost	0.76	0.72	0.82 (0.78–0.86)	0.69
LightGBM	0.70	0.74	0.79 (0.75–0.83)	0.65
XGBoost	0.73	0.69	0.80 (0.76–0.84)	0.66
CatBoost	0.72	0.73	0.80 (0.76–0.84)	0.66

### 3.7 Classification balance across sensitivity and specificity

The balance between sensitivity and specificity was also favorable. AdaBoost achieved specificity of 0.76, sensitivity of 0.72, and a positive predictive value (PPV) of 0.69, representing the strongest combined classification profile among the tested models. LightGBM showed the highest sensitivity at 0.74, but this came with lower specificity at 0.70 and a PPV of 0.65. CatBoost also performed well, with specificity of 0.72, sensitivity of 0.73, and PPV of 0.66. XGBoost showed specificity of 0.73, sensitivity of 0.69, and PPV of 0.66, while LASSO achieved specificity of 0.72, sensitivity of 0.68, and PPV of 0.64. Taken together, these findings indicate that the ensemble methods offered incremental predictive advantage over the penalized linear baseline, with AdaBoost emerging as the best overall classifier in this cohort.

### 3.8 Clinical utility by decision curve analysis

**Figure 3:**
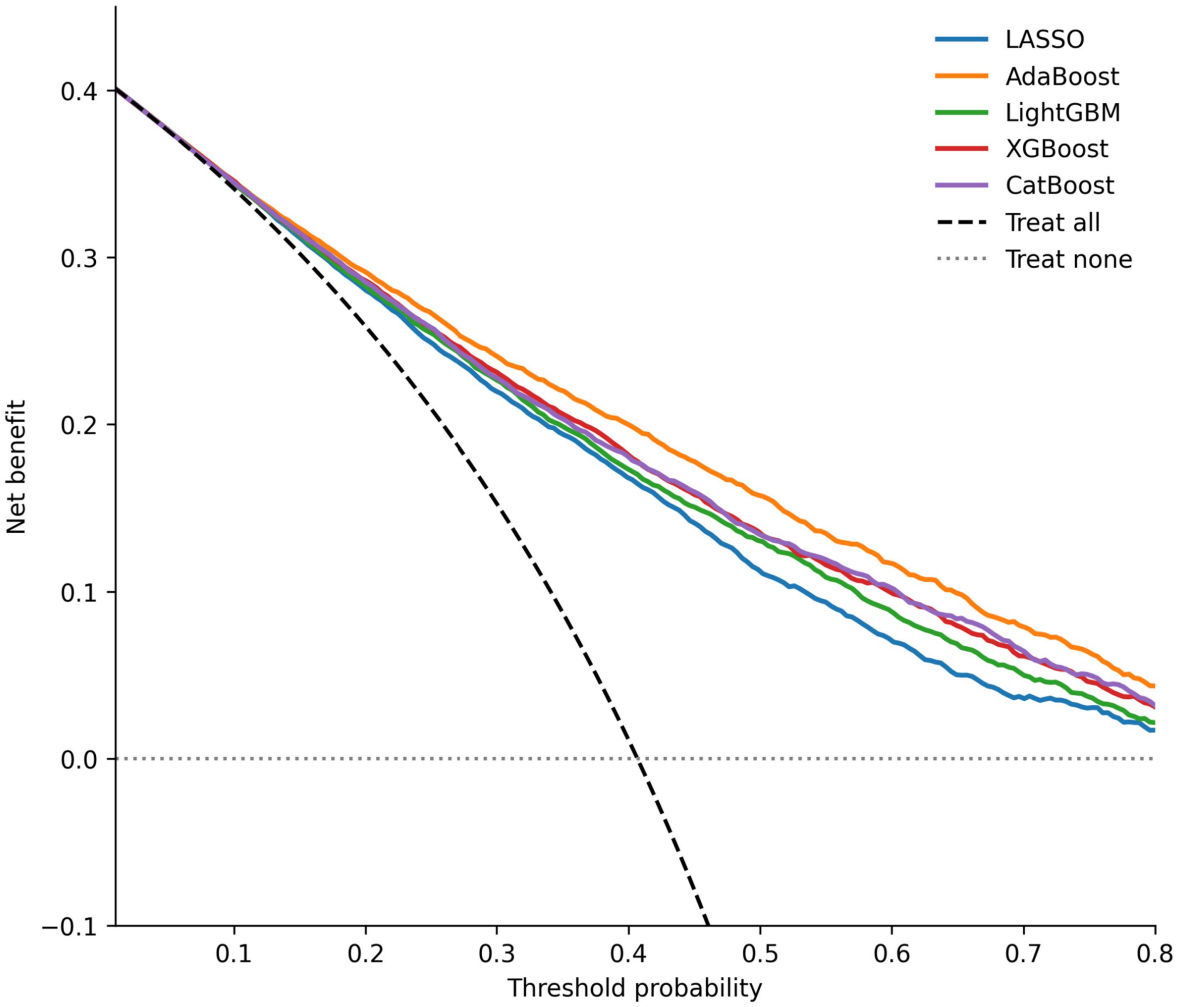
Decision curve analysis of the five prediction models.

Decision curve analysis demonstrated that all candidate models provided clinical net benefit over the treat-none strategy within a clinically relevant threshold range. In general, the model curves also exceeded the treat-all strategy at moderate and higher thresholds, indicating that model-guided decision making would be preferable to applying uniform intervention to all patients. AdaBoost maintained the highest net benefit across much of the displayed threshold range, while the remaining ensemble approaches and LASSO also provided positive net benefit over wide threshold intervals (Figure 3).

### 3.9 Boruta ranking and top-feature confirmation

**Figure 4:**
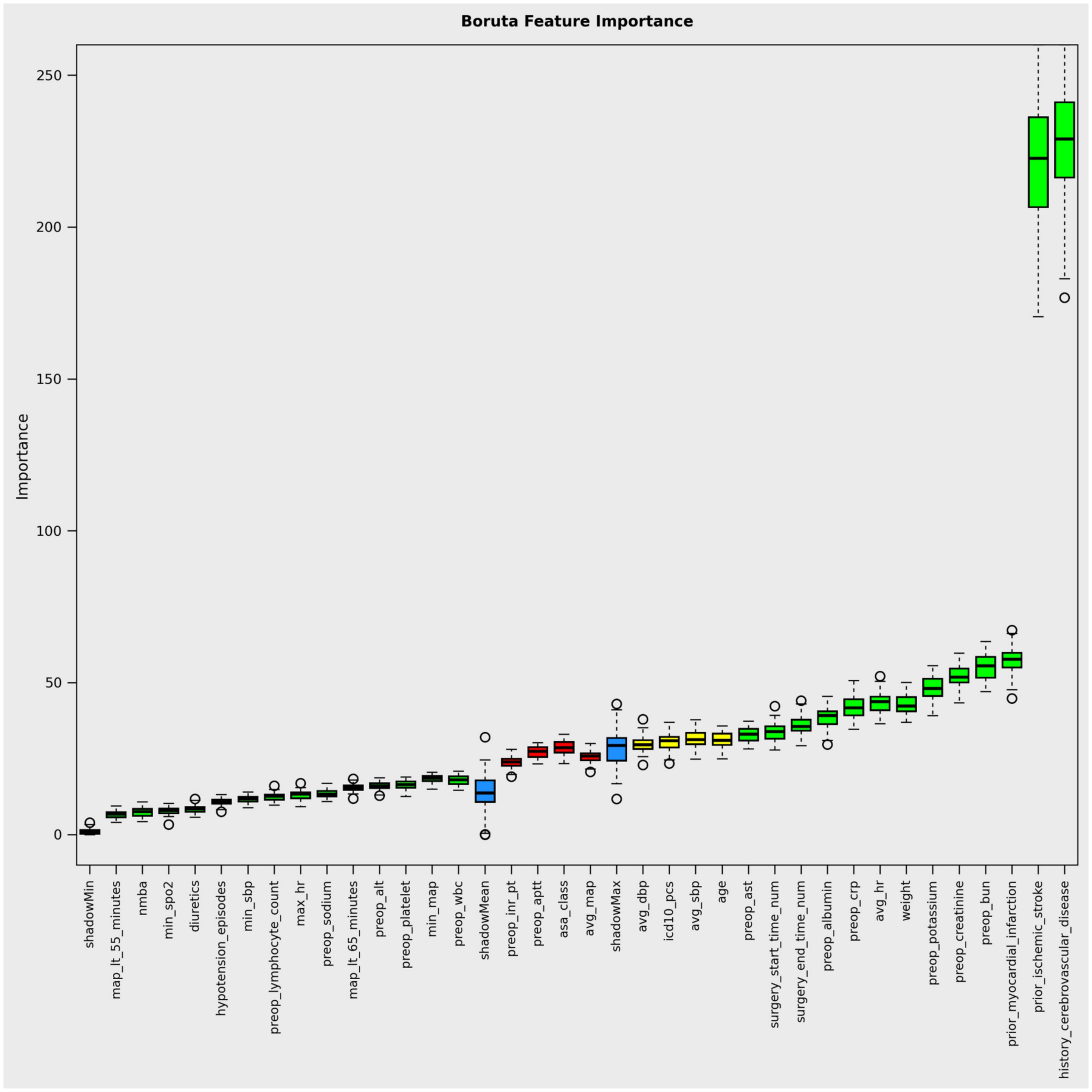
Boruta feature-importance ranking of candidate predictors.

**Figure 5:**
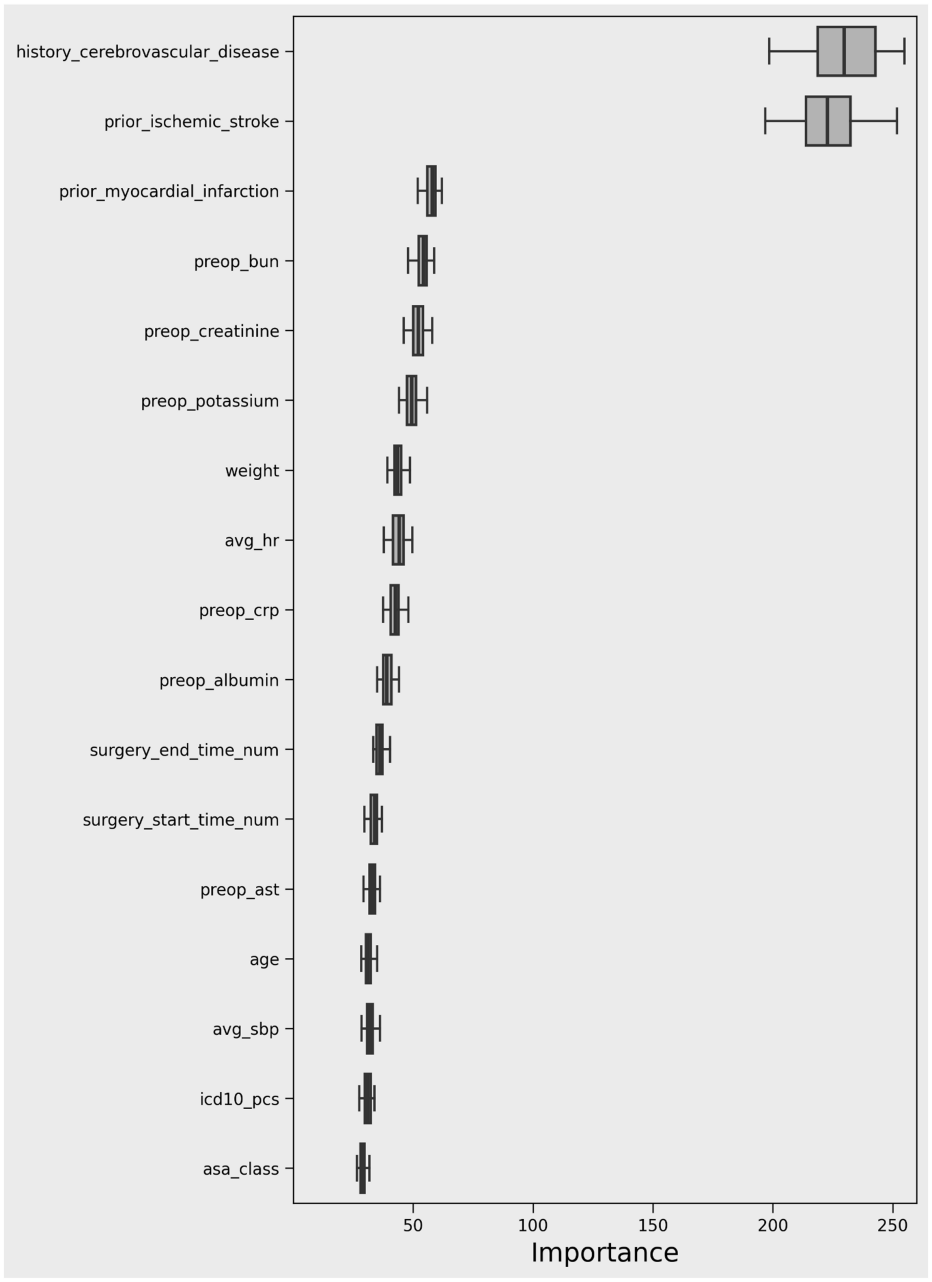
Boruta top confirmed features in the impaired-outcome model.

Boruta feature selection highlighted a set of predictors spanning comorbidity history, intraoperative instability, laboratory status, and perioperative treatment features. The full Boruta importance plot (Figure 4) showed that cerebrovascular history, prior ischemic stroke, prior myocardial infarction, renal-function markers, inflammatory markers, heart-rate summaries, blood-pressure summaries, and several perioperative variables contributed meaningfully to the overall feature hierarchy. The condensed top-feature plot (Figure 5) further emphasized history of cerebrovascular disease and prior ischemic stroke as the most dominant attributes, followed by prior myocardial infarction, preoperative BUN, creatinine, potassium, weight, average heart rate, preoperative C-reactive protein, albumin, surgery timing variables, age, average systolic blood pressure, and ASA class.

### 3.10 Global SHAP structure and dominant predictors

**Figure 6:**
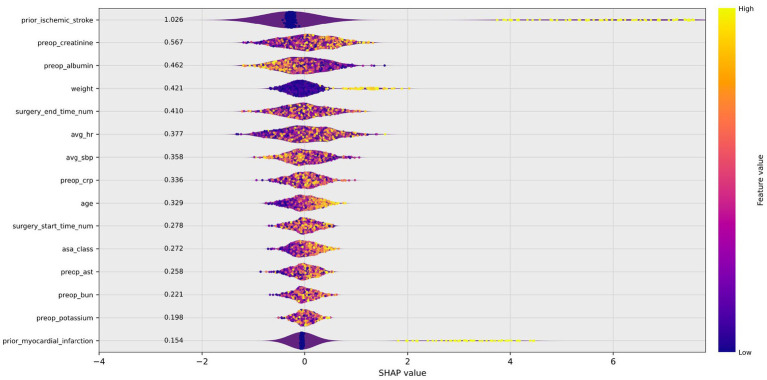
SHAP summary plot of global feature contributions.

The SHAP summary plot in Figure 6 provided a more detailed view of the drivers of model output. Prior ischemic stroke had the largest mean absolute SHAP value, followed by preoperative creatinine, preoperative albumin, weight, surgery end time, average heart rate, average systolic blood pressure, preoperative C-reactive protein, age, surgery start time, ASA class, preoperative AST, preoperative BUN, preoperative potassium, and prior myocardial infarction. Higher creatinine and inflammatory burden tended to push predictions toward higher risk, whereas higher albumin generally shifted predictions toward lower risk, supporting a model that is not only statistically useful but also clinically interpretable.

### 3.11 Integrated interpretation of the modeling results

Taken together, the results suggest that impaired postoperative cardiovascular and cerebrovascular outcome in diabetic patients undergoing non-cardiac surgery is driven by a combination of pre-existing vascular disease, reduced physiologic reserve, adverse laboratory profile, and intraoperative hemodynamic stress. The models did not appear to rely primarily on obscure coding artifacts; rather, they consistently emphasized clinically plausible factors such as prior ischemic stroke, renal dysfunction, albumin level, inflammatory status, age, ASA class, and hypotension burden. In practical terms, the combined information from Appendix Table A1, Table 1, and Figures 1–6 supports the view that ensemble learning can improve predictive performance while still preserving meaningful clinical interpretability.

## 4 Discussion

The principal finding of this analysis is that impaired outcome in diabetic patients undergoing non-cardiac surgery was not explained by a single perioperative insult; instead, it emerged from an interlocking pattern of pre-existing vascular disease, reduced laboratory reserve, inflammatory activation, and intraoperative physiologic stress. This integrated signal was visible at three different levels of the analysis. First, the descriptive table showed that patients with impaired outcome were older, lighter, had higher ASA class, and carried a substantially heavier burden of hypertension, dyslipidemia, prior stroke, prior myocardial infarction, heart failure, arrhythmia, peripheral vascular disease, and renal insufficiency. Second, the physiologic table suggested that hypotension burden rather than isolated oxygen saturation values separated the groups most clearly. Third, the model-interpretation outputs concentrated importance in variables such as prior ischemic stroke, creatinine, albumin, age, inflammatory markers, blood-pressure summaries, and prior myocardial infarction. The convergence of these three analytic levels strengthens the credibility of the overall signal and suggests that the model learned clinically coherent structure rather than spurious noise.

A second major observation is that ensemble learning delivered modest but consistent gains over the sparse penalized linear benchmark. In the harmonized tabulated results, AdaBoost achieved the highest area under the curve, while XGBoost, CatBoost, and LightGBM also slightly outperformed LASSO. This pattern is not surprising. Penalized logistic regression remains a valuable baseline because it is transparent, robust, and parsimonious, but it assumes that covariates act through relatively simple functional forms unless nonlinearity and interaction terms are engineered in advance. By contrast, boosting- and tree-based methods can capture clinically realistic threshold behavior: for example, the fact that risk may rise disproportionately once creatinine crosses a certain level, or that the implications of hypotension depend on age, prior stroke, and baseline comorbidity burden simultaneously (40–49). The practical implication is not that conventional statistics should be abandoned, but that diabetic perioperative data may contain nonlinear structure that ensemble methods are better positioned to exploit.

These findings also fit well with the broader literature on perioperative risk prediction. Legacy tools such as the RCRI and NSQIP calculators remain foundational because of their simplicity and broad recognition, but they deliberately trade granularity for bedside usability (5–8). More recent perioperative studies have shown that richer data streams, including biomarkers, coded comorbidity embeddings, waveform inputs, and large-scale electronic health record features, can improve risk estimation (9, 10, 40–48). Our results sit squarely within that transition. The current model did not rely on exotic variables unavailable to everyday practice; it drew much of its predictive power from variables already familiar to perioperative clinicians, including age, ASA class, renal markers, albumin, inflammatory burden, and blood-pressure instability. That is encouraging because it implies a realistic pathway toward implementation: the barrier is less about inventing new tests than about integrating already-available data more intelligently.

The prominence of prior ischemic stroke, prior myocardial infarction, and history of cerebrovascular disease deserves special attention. These variables dominated both Boruta and SHAP outputs and are biologically plausible anchors of perioperative vulnerability. Diabetes accelerates diffuse atherosclerosis and small-vessel disease, and prior ischemic events signal that these processes have already crossed the threshold from risk factor burden into manifest end-organ injury. A prior stroke may indicate impaired cerebral reserve, advanced systemic vascular disease, atrial arrhythmia exposure, or shared inflammatory and thrombotic mechanisms that continue to shape postoperative risk long after the acute event has resolved. Likewise, prior myocardial infarction and coronary disease reflect fixed scar burden, impaired reserve, and often a more complex medication and revascularization history. These features are therefore not merely historical diagnoses; they are compressed summaries of long-term vascular injury, which helps explain why they retain such strong predictive value even after other covariates are added (3, 4, 11–17, 24–31).

Renal and nutritional markers were equally informative. Preoperative creatinine and blood urea nitrogen ranked highly, consistent with the established link between chronic kidney dysfunction and adverse perioperative cardiovascular outcomes. Kidney dysfunction is both a marker and a mediator: it reflects diffuse vascular disease and impaired homeostatic reserve while also amplifying susceptibility to volume shifts, drug toxicity, acid–base perturbation, and neurohormonal activation. The strong inverse contribution of albumin is also clinically important. Hypoalbuminemia is often interpreted as a nutritional marker, but in perioperative populations it more accurately represents a composite signal of frailty, chronic inflammation, illness severity, and reduced physiologic resilience (37, 38). In diabetic surgical patients, a low albumin level may further indicate coexistence of nephropathy, hepatic dysfunction, catabolic stress, or poor chronic disease control. The model’s emphasis on creatinine, BUN, and albumin therefore suggests that baseline organ reserve may be at least as important as the immediate surgical trigger in determining who decompensates after non-cardiac surgery.

Inflammatory and metabolic signals added another layer of meaning. C-reactive protein, white blood cell count, platelet count, AST, and potassium all contributed either in the descriptive table or interpretability outputs. None of these variables is specific to cardiovascular injury, but together they describe the biologic terrain on which postoperative events occur. Elevated CRP and leukocytosis may reflect chronic low-grade inflammation, acute intercurrent illness, occult infection, or tissue stress; higher platelet counts can index reactive inflammation or altered thrombotic potential. AST may partly capture hepatic reserve or systemic injury, whereas potassium abnormalities often signal renal dysfunction, medication effects, and arrhythmic vulnerability. Importantly, these markers are consistent with modern views of perioperative myocardial injury, which emphasize that postoperative cardiovascular events are not solely plaque-rupture phenomena but often arise from supply–demand mismatch, inflammation, anemia, hypotension, tachycardia, and underlying vascular susceptibility (25–31). The present model appears to align with that pathophysiologic pluralism rather than reducing risk to a single mechanism.

The hemodynamic findings may be among the most actionable results. The impaired group experienced more hypotension episodes and longer durations below clinically relevant MAP thresholds, and the Boruta ranking also retained multiple blood-pressure and hypotension features. This echoes a robust literature linking intraoperative hypotension with myocardial injury, acute kidney injury, major adverse cardiac events, and mortality after non-cardiac surgery (32–36). From a translational perspective, hypotension is more immediately modifiable than a prior stroke history or chronic renal impairment. It is therefore notable that the model simultaneously highlighted immutable historical risk and potentially preventable physiologic stress. That combination is exactly what clinicians need: stable preoperative features to identify who is vulnerable, and perioperative management features to identify where intervention may help. For diabetic patients, who may have impaired autonomic compensation and reduced end-organ reserve, avoiding prolonged or deep hypotension may be especially important. Future work should test whether integrating such models into intraoperative monitoring workflows can guide more individualized blood-pressure targets or earlier rescue strategies.

Medication variables should be interpreted carefully. Beta-blockers, statins, calcium-channel blockers, insulin, anticoagulants, antiplatelet agents, diuretics, antifibrinolytics, and proton-pump inhibitors were all more common in the impaired group. It would be incorrect to read these associations as simple drug harm. In observational perioperative datasets, medication exposure usually functions as a proxy for the disease complexity that prompted the prescription in the first place. A patient taking insulin, antiplatelet therapy, and diuretics likely differs systematically from one taking none of them, even before the operation begins. This is why explainable machine-learning studies must distinguish predictive importance from causal effect. In the current setting, medication features are valuable because they carry hidden information about cardiovascular burden, prior events, clinician concern, and perioperative management intensity. Their predictive contribution therefore complements, rather than replaces, the direct clinical variables (40–49).

An important strength of the present work is the deliberate combination of discrimination analysis, feature selection, and explainability. Too many predictive studies stop after reporting the AUC, which is necessary but insufficient. A model with reasonable discrimination may still be clinically useless if it offers little net benefit at relevant thresholds or if its most influential features are implausible, unstable, or unavailable in practice. By combining tabulated classification metrics, decision-curve reasoning, Boruta screening, and SHAP explanation, the current analysis provides a fuller account of model value (50–52). This matters for manuscript quality and for real implementation. A perioperative risk model is more likely to be adopted when clinicians can verify that it privileges recognizable risk domains—vascular history, renal reserve, inflammatory status, and hemodynamic instability—rather than arbitrary coding artifacts. In this study, the explainability outputs were reassuring in that core clinical variables dominated the feature hierarchy.

Several limitations should be acknowledged. First, the study is retrospective, so associations should not be interpreted as causal effects. Second, the outcome was defined as a database-derived composite impaired outcome incorporating major adverse cardiovascular and cerebrovascular events and related cardiovascular complications; although pragmatic, this endpoint is heterogeneous and the present materials did not include adjudicated component-event frequencies, component-specific sensitivity analyses, or a recoverable record of the exact follow-up duration was not available in the finalized analytic package. Third, the current analytic package did not provide a full reproducible record of cohort assembly, diabetes ascertainment, missing-data handling, class-imbalance strategy, hyperparameter tuning, threshold optimization, calibration statistics, or external validation, so the model’s probability accuracy, transportability, and bedside readiness remain uncertain. Fourth, some appendix variables were structurally zero or non-informative in the finalized extract and were retained only for reporting completeness. Fifth, medication variables and coded procedure indicators may encode care processes or institutional practice patterns that shift across settings. These are common issues in perioperative machine-learning research and underscore why future validation across hospitals, procedure categories, and calendar periods is essential (38–47).

The diabetes-specific findings are also worth emphasizing because the model appears to have captured more than glycemia alone. Insulin use, cardiovascular medications, renal markers, and inflammatory variables together suggest that diabetes contributed to risk partly as a marker of systemic disease severity rather than merely as a binary diagnosis. This is consistent with prior literature showing that perioperative hyperglycemia, insulin-treated diabetes, and elevated HbA1c can each identify different facets of surgical vulnerability (16–23). Some patients are chronically adapted to hyperglycemia but carry profound vascular injury; others present with stress hyperglycemia that may reflect acute inflammatory or neuroendocrine activation. A sophisticated perioperative model should not force these distinct states into a single explanatory channel. The current analysis moves in that direction by embedding glucose-related treatment and end-organ manifestations within a broader cardiovascular phenotype. From a manuscript perspective, this helps justify why diabetic status should be treated as a context requiring tailored modeling rather than a covariate to be merely adjusted away.

At the current stage, the clinical implications of this work should be interpreted cautiously. The present results support hypothesis generation and internal comparative modeling rather than immediate bedside deployment. In future studies, a prospective framework could examine whether baseline risk estimates combined with intraoperative physiologic updates might help identify patients who merit closer postoperative surveillance. Any such application, however, should follow calibration assessment, external validation, and explicit evaluation of how threshold probabilities map to concrete clinical actions (50).

These findings should also be interpreted against the contemporary global burden of diabetes. Recent international estimates indicate that 589 million adults were living with diabetes in 2024 and that the global total may rise to 853 million by 2050 (1). Diabetes was responsible for an estimated 3.4 million deaths in 2024 (2), which further underscores the importance of more refined perioperative risk stratification in vulnerable surgical populations.

Even with these constraints, the study has several clear strengths. It focuses on a clinically important but under-modeled population, uses a reasonably large diabetic surgical cohort, compares multiple algorithms rather than declaring a single model *a priori*, and incorporates clinically interpretable explainability outputs. The findings also generate concrete hypotheses for future work. For example, it will be important to determine whether a simplified perioperative diabetic risk tool built around prior vascular events, creatinine, albumin, CRP, ASA class, and hypotension burden can recover most of the performance of more complex models, and whether such a tool remains accurate after calibration testing and external validation (3, 4, 24–26, 50).

## 5 Conclusion

In diabetic patients undergoing non-cardiac surgery, impaired outcome was associated with pre-existing vascular disease, reduced renal and nutritional reserve, inflammatory activation, and intraoperative hemodynamic instability. Ensemble models, especially AdaBoost, showed modest internal improvement over LASSO, but further calibration testing, external validation, and reproducible pipeline reporting are required before clinical use.

## Data Availability

The INSPIRE analytic extract used for this study is institutionally governed and de-identified. Patient-level data are not publicly available because of privacy and governance restrictions. Requests for additional methodological clarification may be directed to the corresponding author and will be considered subject to institutional approval.
