# Supplementary material for: Machine-learning prediction of impaired outcome in diabetic patients undergoing non-cardiac surgery
Source: Front Med (Lausanne). 2026 Jun 5;13:1846842. doi: 10.3389/fmed.2026.1846842 (PMC13279090; doi:10.3389/fmed.2026.1846842)
Supplement: Supplementary file 1 [file Table_1.DOCX]

Supplementary Material

# Supplementary Tables

Appendix A. Baseline characteristics and perioperative variables

Table A1. Baseline characteristics, intraoperative physiologic parameters, preoperative laboratory tests, and cardiovascular medication use in diabetic patients undergoing non-cardiac surgery, stratified by outcome status.

| **Variable** | **In all (N=4293)** | **Control (n=3176)** | **Impaired (n=1117)** | ***P*-value** |
| --- | --- | --- | --- | --- |
| age | 70.0 [60.0–75.0] | 65.0 [60.0–75.0] | 70.0 [65.0–75.0] | <0.001 |
| weight | 65.0 [55.0–70.0] | 65.0 [55.0–70.0] | 60.0 [50.0–65.0] | <0.001 |
| asa_class | 2.0 [2.0–2.0] | 2.0 [2.0–2.0] | 2.0 [2.0–3.0] | <0.001 |
| Anesthesia type: MAC | 1 117 (26.0%) | 895 (28.2%) | 222 (19.9%) | <0.001 |
| Anesthesia type: General | 2 785 (64.9%) | 1 982 (62.4%) | 803 (71.9%) | <0.001 |
| Anesthesia type: Local | 391 (9.1%) | 299 (9.4%) | 92 (8.2%) | 0.246 |
| hypertension | 3 237 (75.4%) | 2 306 (72.6%) | 931 (83.4%) | <0.001 |
| dyslipidemia | 2 026 (47.2%) | 1 426 (44.9%) | 600 (53.7%) | <0.001 |
| carotid_stenosis | 97 (2.3%) | 59 (1.9%) | 38 (3.4%) | 0.003 |
| valvular_heart_disease | 221 (5.1%) | 143 (4.5%) | 78 (7.0%) | 0.001 |
| atrial_fibrillation | 232 (5.4%) | 137 (4.3%) | 95 (8.5%) | <0.001 |
| copd | 204 (4.8%) | 124 (3.9%) | 80 (7.2%) | <0.001 |
| renal_insufficiency | 245 (5.7%) | 147 (4.6%) | 98 (8.8%) | <0.001 |
| hepatic_insufficiency | 34 (0.8%) | 23 (0.7%) | 11 (1.0%) | 0.275 |
| prior_ischemic_stroke | 399 (9.3%) | 271 (8.5%) | 128 (11.5%) | 0.002 |
| prior_myocardial_infarction | 220 (5.1%) | 139 (4.4%) | 81 (7.3%) | <0.001 |
| history_cerebrovascular_disease | 541 (12.6%) | 362 (11.4%) | 179 (16.0%) | <0.001 |
| history_coronary_artery_disease | 902 (21.0%) | 595 (18.7%) | 307 (27.5%) | <0.001 |
| history_heart_failure | 307 (7.2%) | 186 (5.9%) | 121 (10.8%) | <0.001 |
| history_arrhythmia | 423 (9.9%) | 262 (8.3%) | 161 (14.4%) | <0.001 |
| history_peripheral_vascular_disease | 94 (2.2%) | 57 (1.8%) | 37 (3.3%) | 0.002 |
| Minimum SpO₂ (%) | 94 [85–95] | 94 [85–95] | 94 [85–95] | <0.001 |
| Hypoxia episodes, n | 0 [0–1] | 0 [0–1] | 0 [0–2] | <0.001 |
| SpO₂ <90% duration (min) | 0 [0–0] | 0 [0–0] | 0 [0–0] | 0.029 |
| SpO₂ <85% duration (min) | 0 [0–0] | 0 [0–0] | 0 [0–0] | 1 |
| Hypotension episodes, n | 0 [0–1] | 0 [0–1] | 0 [0–2] | <0.001 |
| MAP <65 duration (min) | 0 [0–0] | 0 [0–0] | 0 [0–1] | <0.001 |
| MAP <55 duration (min) | 0 [0–0] | 0 [0–0] | 0 [0–0] | <0.001 |
| Hypoxia <90%, n (%) | 1 318 (30.7%) | 953 (30.0%) | 365 (32.7%) | 0.104 |
| Severe hypoxia <85%, n (%) | 0 (0.0%) | 0 (0.0%) | 0 (0.0%) | 1 |
| MAP <65 mmHg, n (%) | 1 521 (35.4%) | 1 041 (32.8%) | 480 (43.0%) | <0.001 |
| MAP <55 mmHg (severe), n (%) | 215 (5.0%) | 76 (2.4%) | 139 (12.4%) | <0.001 |
| Preoperative WBC (×10⁹/L) | 9.5 [6.7–13.8] | 8.9 [6.3–12.2] | 12.2 [7.9–16.5] | <0.001 |
| Preoperative platelets (×10⁹/L) | 258 [200–347] | 258 [189–314] | 273 [211–407] | <0.001 |
| beta_blocker_use | 587 (13.7%) | 342 (10.8%) | 245 (21.9%) | <0.001 |
| acei_use | 0 (0.0%) | 0 (0.0%) | 0 (0.0%) | 1 |
| arb_use | 0 (0.0%) | 0 (0.0%) | 0 (0.0%) | 1 |
| statin_use | 1 143 (26.6%) | 767 (24.1%) | 376 (33.7%) | <0.001 |
| ccb_use | 1 079 (25.1%) | 683 (21.5%) | 396 (35.5%) | <0.001 |
| insulin_use | 1 192 (27.8%) | 745 (23.5%) | 447 (40.0%) | <0.001 |
| anticoagulant_use | 243 (5.7%) | 138 (4.3%) | 105 (9.4%) | <0.001 |
| antiplatelet_use | 1 112 (25.9%) | 703 (22.1%) | 409 (36.6%) | <0.001 |
| diuretics | 462 (10.8%) | 265 (8.3%) | 197 (17.6%) | <0.001 |
| inhalational_general | 2 785 (64.9%) | 1 982 (62.4%) | 803 (71.9%) | <0.001 |
| iv_general | 1 117 (26.0%) | 895 (28.2%) | 222 (19.9%) | <0.001 |
| local_anesthetic | 391 (9.1%) | 299 (9.4%) | 92 (8.2%) | 0.246 |
| opioid | 4 285 (99.8%) | 3 170 (99.8%) | 1 115 (99.8%) | 1 |
| sedative | 4 287 (99.9%) | 3 170 (99.8%) | 1 117 (100.0%) | 0.182 |
| nmba | 4 153 (96.7%) | 3 068 (96.6%) | 1 085 (97.1%) | 0.426 |
| reversal | 275 (6.4%) | 189 (6.0%) | 86 (7.7%) | 0.047 |
| antibiotic | 4 257 (99.2%) | 3 146 (99.1%) | 1 111 (99.5%) | 0.268 |
| antifibrinolytic | 2 044 (47.6%) | 1 378 (43.4%) | 666 (59.6%) | <0.001 |
| ppi | 3 747 (87.3%) | 2 727 (85.9%) | 1 020 (91.3%) | <0.001 |

**Note.** Patients were divided into a Control group (no impaired outcome) and an Impaired group, defined as those who experienced major adverse cardiovascular and cerebrovascular events and/or related adverse cardiovascular outcomes during follow-up. Continuous variables are reported as median [interquartile range], and categorical variables as number (percentage). Between-group comparisons were performed using the Mann–Whitney U test for continuous variables and the χ² test or Fisher’s exact test for categorical variables, as appropriate.
